# Association of Multimorbidity, Disease Clusters, and Modification by Genetic Factors With Risk of Dementia

**DOI:** 10.1001/jamanetworkopen.2022.32124

**Published:** 2022-09-20

**Authors:** Catherine M. Calvin, Megan C. Conroy, Sarah F. Moore, Elżbieta Kuźma, Thomas J. Littlejohns

**Affiliations:** 1 Nuffield Department of Population Health, University of Oxford, Oxford, United Kingdom; 2 College of Medicine and Health, University of Exeter, Exeter, United Kingdom; 3 Albertinen-Haus Centre for Geriatrics and Gerontology, University of Hamburg, Hamburg, Germany

## Abstract

**Importance:**

Individual conditions have been identified as risk factors for dementia; however, it is important to consider the role of multimorbidity, as conditions often co-occur.

**Objective:**

To investigate whether multimorbidity is associated with incident dementia and whether associations vary by different clusters of disease and genetic risk for dementia.

**Design, Setting, and Participants:**

This population-based prospective cohort study used data from the UK Biobank cohort, with baseline data collected between 2006 and 2010 and with up to 15 years of follow-up. Participants included women and men without dementia and aged at least 60 years at baseline. Medical conditions were captured as part of nurse-led verbal interviews conducted at baseline assessment centers. Data were analyzed from October 2020 to July 2022.

**Exposures:**

The presence of at least 2 long-term conditions from a preselected list of 42 conditions was used to define multimorbidity. High genetic risk for dementia was based on presence of 1 or 2 apolipoprotein (APOE) ε4 alleles.

**Main Outcomes and Measures:**

The main outcome, incident dementia, was derived from hospital inpatient and death registry records. Associations of multimorbidity with dementia were assessed with Cox proportional hazards models.

**Results:**

A total of 206 960 participants (mean [SD] age, 64.1 [2.9] years, 108 982 [52.7%] women) were included in the final sample, of whom 89 201 participants (43.1%) had multimorbidity. Over a mean (SD) of 11.8 (2.2) years of follow-up, 6182 participants (3.0%) developed dementia. The incidence rate was 1.87 (95% CI, 1.80-1.94) per 1000 person-years for those without multimorbidity and 3.41 (95% CI, 3.30-3.53) per 1000 person-years for those with multimorbidity. In Cox proportional hazards models adjusted for age, sex, ethnicity, education, socioeconomic status, and APOE-ε4 carrier status, multimorbidity was associated with an increased risk of incident dementia (hazard ratio [HR], 1.63 [95% CI, 1.55-1.71]). The highest dementia risk was observed for the hypertension, diabetes, and coronary heart disease cluster (HR, 2.20 [95% CI, 1.98-2.46]) and pain, osteoporosis, and dyspepsia cluster (HR, 2.00 [95% CI, 1.68-2.37]) in women and in the diabetes and hypertension cluster (HR, 2.24 [95% CI, 1.97-2.55]) and coronary heart disease, hypertension, and stroke cluster (HR, 1.94 [95% CI, 1.71-2.20]) in men, compared with no multimorbidity. The associations between multimorbidity and dementia were greater in those with a lower genetic risk of dementia (HR, 1.96 [95% CI, 1.81-2.11]) than in those with a higher genetic risk of dementia (HR, 1.39 [95% CI, 1.30-1.49]). Similar findings were observed when stratifying diseases clusters by genetic risk for dementia.

**Conclusions and Relevance:**

These findings suggest that multimorbidity was associated with an increased risk of dementia. The associations varied by clusters of disease and genetic risk for dementia. These findings could help with the identification of individuals at high risk of dementia as well as the development of targeted interventions to reduce or delay dementia incidence.

## Key Points

**Question:**

Is multimorbidity associated with risk of incident dementia?

**Findings:**

In this cohort study of 206 960 participants, multimorbidity was associated with a 63% increased risk of incident dementia over 15 years of follow-up. The risk of dementia associated with multimorbidity was highest in individuals with cardiovascular and cardiometabolic clusters of disease and those with the lower genetic risk of dementia.

**Meaning:**

These findings suggest that disease clusters might be important for dementia risk stratification and the development of targeted interventions to prevent or delay dementia.

## Teaser

This cohort study investigates whether multimorbidity is associated with incident dementia and whether associations differ by different clusters of disease and genetic risk of dementia.

## Introduction

Owing to increasing life expectancy, the global prevalence of dementia is projected to increase 3-fold within 30 years, from 57 million to 153 million individuals by 2050.^1^ Despite the increasing overall incidence, there is evidence that the age-specific prevalence of dementia is declining.^2^ This decline in prevalence could partially be due to improvements in the control and treatment of cardiovascular and metabolic diseases, such as hypertension, stroke, and diabetes.^3^ These and other health-related conditions have previously been identified as potentially key modifiable risk factors for dementia reduction.^4,5^

However, health conditions often do not occur in isolation, with approximately one-third of the global population living with 2 or more long-term health conditions, termed *multimorbidity*.^6,7^ Similar to dementia, the prevalence of multimorbidity increases substantially with age, with approximately two-thirds of individuals aged 65 to 84 years and more than 80% of those aged 85 years or older living with multiple conditions.^8^ Consequently, when investigating the role of certain health conditions as risk factors for dementia, it is important to understand the role of multiple conditions, in particular whether certain clusters of disease are differentially associated with dementia risk.^9^

To our knowledge, 2 studies have investigated the association between multimorbidity and dementia risk.^10,11^ Both found that multimorbidity was associated with an increased risk of dementia, while one found that neuropsychiatric, cardiovascular, and sensory impairment or cancer clusters, but not a respiratory, metabolic, and musculoskeletal cluster, were associated with an increased risk.^11^ The same study found no evidence that apolipoprotein (APOE) ε4, the strongest genetic risk factor for dementia,^12^ modified these associations.^11^

However, these studies included between 500 and 700 participants with incident dementia and might have lacked sufficient statistical power to detect whether certain disease clusters are associated with different risks of dementia and whether genetic risk for dementia modifies these associations.^10,11^

In this study of more than 200 000 participants, including nearly 6200 participants with incident dementia, we investigated whether multimorbidity and disease clusters were associated with incident dementia and whether any observed associations were modified by genetic risk for dementia.

## Methods

The UK Biobank received ethical approval from the National Health Service North West Centre for Research Ethics Committee. All participants provided electronically signed informed consent. This study is reported following the Strengthening the Reporting of Observational Studies in Epidemiology (STROBE) reporting guideline.

### Population

Between 2006 and 2010, approximately half a million women and men aged between 40 and 69 years joined the UK Biobank study.^13,14^ All participants attended 1 of 22 baseline assessment centers located throughout England, Scotland, and Wales and provided informed consent. At assessment, participants provided sociodemographic, lifestyle, and health-related information through a touchscreen questionnaire and nurse-led verbal interview and underwent a range of physical examinations. Blood samples were obtained and used to perform genome-wide genotyping at a later date.

A total of 502 412 individuals participated in UK Biobank. To restrict to individuals at risk of developing dementia over the follow-up period, we excluded 284 943 participants aged younger than 60 years at baseline. A further 166 participants with prevalent dementia and 10 343 participants with missing covariate information were excluded, resulting in a final sample of 206 960 participants.

### Multimorbidity

Participants self-reported medical conditions during the nurse-led verbal interview at baseline assessment. The interviewer was guided by a tree structure loosely based on the *International Statistical Classification of Diseases and Related Health Problems, Tenth Revision (ICD-10)* coding system to ensure entries were standardized.^15^ In this study, the diseases included in the multimorbidity definition were based on the list developed by Barnett and colleagues (eTable 1 in the Supplement).^8,16^ Multimorbidity was defined as the presence of at least 2 of the 42 conditions. Participants with zero or 1 condition were defined as not having multimorbidity and formed the reference group in the analyses.

### Dementia

Dementia was ascertained using hospital inpatient and death registry records. Primary and secondary hospital diagnoses and causes of deaths were recorded using the *International Classification of Diseases, Ninth Revision *(*ICD-9*) and *ICD-10* coding systems. The codes for dementia were previously selected and validated by the UK Biobank outcome adjudication group and are listed in eTable 2 in the Supplement.^17^ Participants with a diagnosis of dementia prior to study baseline or who self-reported having dementia during the verbal interview at baseline assessment were excluded from this study.

### Covariates

Sociodemographic covariates included age in years, sex (women and men), ethnicity (classified based on self report and dichotomized as White and non-White), education (college or university or professional qualification, secondary school or vocational qualification, and no qualification), and tertiles of socioeconomic status (measured with the Townsend deprivation score combining information on social class, employment, car availability, and housing).^18^ The non-White ethnicity category included participants who identified as Asian or Asian British, Black or Black British, Chinese, mixed ethnicity, or other ethnic group. Ethnicity was included because the risk of dementia and multimorbidity has been reported to vary substantially by ethnic group.^4,19^ APOE-ε4 carrier status was classified as noncarrier and carrier and determined using the rs429358 and rs7412 single nucleotide polymorphisms, which were directly genotyped on the UK Biobank arrays.^20^

### Statistical Analysis

#### All Analyses

Cox proportional hazards models adjusted for age, sex, ethnicity, education, socioeconomic status, and APOE-ε4 carrier status were used to estimate the association of multimorbidity and disease clusters with incident dementia. Person-years were calculated from date of attending baseline assessment until date of first dementia diagnosis, date of death, date lost to follow-up, or end of follow-up, whichever occurred first. End of follow-up was based on the availability of the medical record data in UK Biobank, which was censored at September 30, 2021, for England; July 31, 2021, for Scotland; and February 28, 2018, for Wales. All models were assessed for the proportionality of hazards assumption using log-log plots, Kaplan-Meier observed survival curves, and post hoc tests of proportional hazards with Schoenfeld residuals. Sex violated the proportional hazards assumption and was adjusted for using a stratified Cox model. Participants with missing data or who answered prefer not to answer or do not know constituted less than 5% of the sample and were excluded from the analyses. Dementia incidence rates (IRs) per 1000 person-years were calculated for each exposure group.

*P* values were 2-sided, and the type I error rate for statistical significance was set at α = .05. Analyses were performed using Stata SE version 17.0 (StataCorp). RStudio version 1.4.1717 (*poLCA* package; R Project for Statistical Computing) was used to derive disease clusters for the latent class analysis. The analyses were performed from October 2020 to July 2022.

#### Multimorbidity

In the main analysis we investigated the association between multimorbidity (≥2 conditions) compared with no multimorbidity (≤1 condition) and incident dementia. In a sensitivity analysis, we repeated the main analysis using 3 separate follow-up periods of up to 5 years, more than 5 years to 10 years and longer than 10 years. This was to explore the potential for reverse causation bias due to dementia’s long prodromal period, whereby the underlying pathological characteristics of preclinical dementia may affect health several years prior to a clinical diagnosis.^21^ In secondary analyses, we investigated the possibility of a dose-response association by categorizing multimorbidity as no more than 1 condition compared with 2, 3, 4, 5, or 6 or more conditions. To investigate potential effect modification by sociodemographic factors, interaction terms were entered into the main model for multimorbidity by age, sex, ethnicity, education, and socioeconomic status, then stratified analyses were performed within the following groups for each characteristic: age (<65 years and ≥65 years), sex (women and men); ethnicity (White and non-White), education (college or university or professional qualification, secondary school or vocational qualification, and no qualification), and socioeconomic status (in tertiles). In a secondary analysis, we investigated the association of multimorbidity with Alzheimer disease and vascular dementia.

#### Disease Clusters

Latent class analysis was used to determine disease clusters, allocating each participant with multimorbidity to a single nonoverlapping cluster while allowing health conditions to contribute by varying probabilities to multiple clusters.^22^ Clusters were estimated separately for men and women, as output from exploratory models of the total sample showing several sex-dominant cluster groups (eTable 3 in the Supplement). A random training sample of 80% of participants with multimorbidity was used to determine the optimal number of clusters in men and women and subsequently to estimate the association of disease clusters with dementia risk. Output statistics were generated for multiple latent class analysis models of between 1 to 12 cluster solutions, and the optimal numbers of clusters were determined using a combination of sample size–adjusted Bayesian Information Criteria statistics, clinical judgement, and capping the smallest cluster to greater than 5% of the training sample. Each cluster within men and women was characterized by the 3 health conditions with the highest probabilities greater than 5% of contributing to that cluster, excluding conditions for which their observed prevalence was equal to or less than that of the expected prevalence of the total population of men and women.

To assess the validity of the determined cluster solutions, conditions from the remaining 20% of men and women with multimorbidity (a test sample) were entered into latent class analysis models, setting the number of clusters to match the optimal number from the training set. The characterization and relative size of the clusters determined from the training and test samples were compared, as were their associations with dementia risk in Cox proportional hazards models.

#### Modification by APOE-ε4

An interaction term for multimorbidity and APOE-ε4 carrier status was first entered into the main models, then stratified analyses were performed within noncarrier and carrier subgroups. These analyses were repeated for each disease cluster. Due to the known variance in APOE status by ethnicity^23^ and the small proportion of participants who reported an ethnicity other than White, these analyses were restricted to those who reported White ethnicity.

## Results

The final sample included 206 960 participants, with an overall mean (SD) age of 64.1 (2.9) years, including 108 982 (52.7%) women. Of these, 89 201 (43.1%) had multimorbidity at baseline. Participants with multimorbidity were more likely to be older, be women, report non-White ethnicity, have lower educational qualifications, and be from more socioeconomically deprived areas, compared with those with no multimorbidity (Table 1). No difference in APOE-ε4 carrier status was observed by presence or absence of multimorbidity. Baseline characteristics by incident dementia are presented in eTable 4 in the Supplement. A total of 6182 participants (3.0%) developed dementia over 2 451 957 person-years of follow-up, with a mean (SD) of 11.8 (2.2) years of follow-up per participant.

**Table 1.** Baseline Characteristics by Multimorbidity

Characteristics	Participants, No. (%)		
	No multimorbidity (n = 117 759)	Multimorbidity (n = 89 201)	Total sample (N = 206 960)
Age, mean (SD), y	63.9 (2.8)	64.5 (2.9)	64.1 (2.9)
Sex			
Women	61 803 (52.5)	47 179 (52.9)	108 982 (52.7)
Men	55 956 (47.5)	42 022 (47.1)	97 978 (47.3)
Ethnic group			
White	114 860 (97.5)	86 311 (96.8)	201 171 (97.2)
Non-White^a^	2899 (2.5)	2890 (3.2)	5789 (2.8)
Education			
College, university, or professional qualification	52 629 (44.7)	34 237 (38.4)	86 866 (42.0)
Secondary school or vocational qualification	36 834 (31.3)	26 845 (30.1)	63 679 (30.8)
No qualification	28 296 (24.0)	28 119 (31.5)	56 415 (27.3)
Socioeconomic status, tertile			
1 (least deprived)	27 304 (23.2)	17 554 (19.7)	44 858 (21.7)
2	73 038 (62.0)	53 738 (60.2)	126 776 (61.3)
3 (most deprived)	17 417 (14.8)	17 909 (20.1)	35 326 (17.1)
APOE-ε4 status			
Noncarrier	84 592 (71.8)	64 121 (71.9)	148 713 (71.9)
Carrier	33 167 (28.2)	25 080 (28.1)	58 247 (28.1)
Follow-up, mean (SD), y	12.0 (2.0)	11.6 (2.4)	11.8 (2.2)

Abbreviation: APOE, apolipoprotein E.

^a^ Non-White ethnicity includes participants who identified as Asian or Asian British, Black or Black British, Chinese, mixed ethnicity, or other ethnic group.

### Multimorbidity

**Figure 1.**
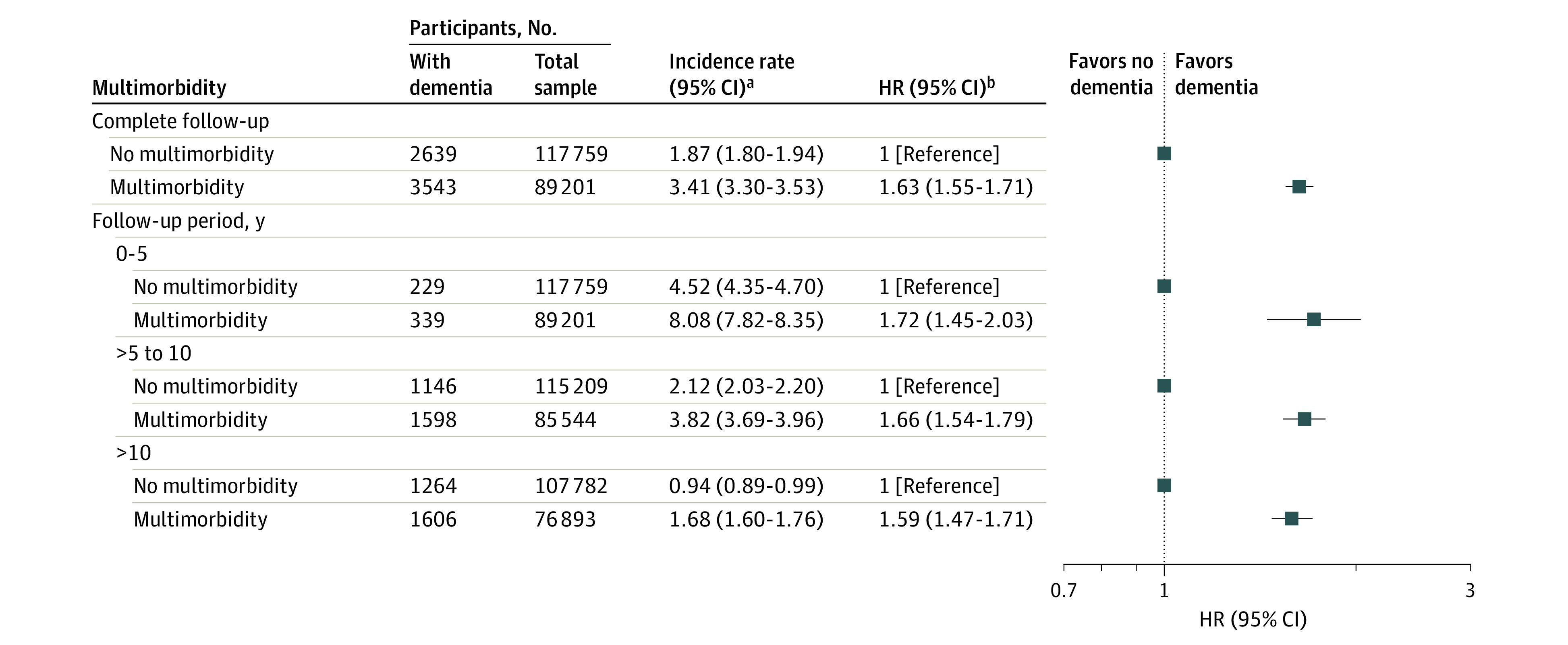
Cox Proportional Hazards Models for the Association Between Multimorbidity and Incident Dementia by Different Follow-up Periods ^a^Incidence rate per 1000 person-years. ^b^All models adjusted for age, ethnicity, education, socioeconomic status, and apolipoprotein ε4 status.

**Figure 2.**
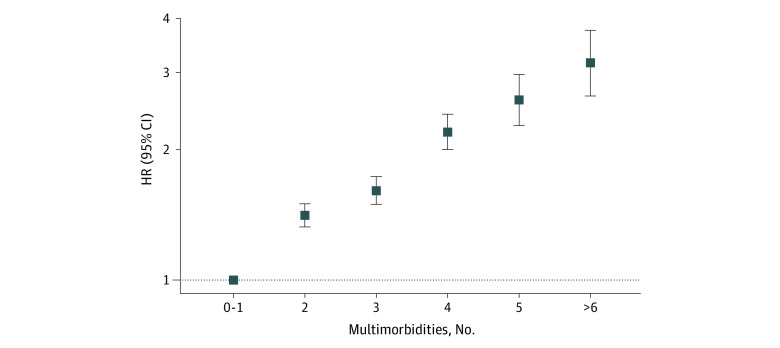
Cox Proportional Hazards Models for the Association Between Number of Multimorbid Conditions and Incident Dementia All models adjusted for age, ethnicity, education, socioeconomic status, and APOE-ε4 status. Dementia cases, sample size, hazard ratios, and incidence rates within each number of mulimorbidities group are presented in eTable 5 in the Supplement.

The IR of dementia was almost double in participants with multimorbidity compared with those with no multimorbidity (IR, 3.41 [95% CI, 3.30-3.53] per 1000 person-years vs IR, 1.87 [95% CI, 1.80-1.94] per 1000 person-years) (Figure 1). In fully adjusted Cox proportional hazards models, multimorbidity was associated with an increased risk of dementia compared with no multimorbidity (hazard ratio [HR], 1.63 [95% CI, 1.55-1.71]) (Figure 1). The pattern of associations remained similar when restricting to different periods of follow-up (Figure 1). For instance, for participants with at least 10 years of follow-up, the risk of incident dementia associated with multimorbidity was 59% higher (HR, 1.59 [95% CI, 1.47-1.71]). When investigating number of conditions with dementia risk, a dose-response association compared with no multimorbidity was observed (2 conditions: HR, 1.41 [95% CI, 1.32-1.50]; 3 conditions: HR, 1.61 [95% CI, 1.49-1.73]; 4 conditions: HR, 2.19 [95% CI, 1.99-2.40]; 5 conditions: HR, 2.59 [95% CI, 2.26-2.96]; ≥6 conditions: HR, 3.15 [95% CI, 2.65-3.75]) (Figure 2; eTable 5 in the Supplement).

There was no statistically significant interaction between multimorbidity and age, sex, ethnicity, education, or socioeconomic status in relation to incident dementia. The effect estimates for the association between multimorbidity and incident dementia within each strata of age, sex, ethnicity, education, and socioeconomic status are provided in eTable 6 in the Supplement. Multimorbidity remained associated with dementia subtypes, with a higher risk observed for vascular dementia (917 participants; HR, 2.57 [95% CI, 2.24-2.96]) and an attenuated association for Alzheimer disease (1786 participants: HR, 1.33 [95% CI, 1.21-1.46])

### Disease Clusters

The analysis for women included 41 conditions (prostate disorders excluded), and the analysis for men included 40 conditions (polycystic ovaries and endometriosis excluded). In a comparison of latent class analysis models, we identified 7 clusters as the optimal number for women and 6 clusters as the optimal number for men (eFigure 1 in the Supplement). A thyroid disease cluster was unique to women, and a coronary heart disease (CHD) and diabetes cluster was unique to men (eTable 7 and eTable 8 in the Supplement). There was high similarity in the characteristics of the cluster groups for men and women in the training samples vs the cluster groups in the test samples (eTable 9 in the Supplement). Certain clusters, while consisting of a range of conditions, were largely characterized by 1 disease, such as cancer in both women and men and thyroid disorders in women (eTable 7 and eTable 8 in the Supplement).

All disease clusters in men and women were associated with an increased risk of dementia compared with no multimorbidity (Table 2). In women, the hypertension, diabetes, and CHD cluster (HR, 2.20 [95% CI, 1.98-2.46]) and pain, osteoporosis, and dyspepsia cluster (HR, 2.00 [95% CI, 1.68-2.37]) were associated with the highest risk of incident dementia compared with no multimorbidity. In men, the diabetes and hypertension cluster (HR, 2.24 [95% CI, 1.97-2.55]) and CHD, hypertension, and stroke cluster (HR, 1.94 [95% CI, 1.71-2.20]) were associated with the highest risk of incident dementia compared with no multimorbidity.

**Table 2.** Sex-Stratified Cox Proportional Hazards Models for the Association Between Disease Clusters and Incident Dementia

Disease clusters^a^	Participants, No.		Median morbidities (IQR)	Hazard ratio (95% CI)^b^	Incident rate (95% CI)^c^
	With incident dementia	Overall			
Women					
No multimorbidity	1231	61 803	1 (0–1)	1 [Reference]	1.64 (1.55-1.74)
Hypertension (100%), Diabetes (23%), and CHD (15%)	452	9107	2 (2-3)	2.20 (1.98-2.46)	4.22 (3.85-4.63)
Cancer (100%)	188	6440	2 (2-3)	1.37 (1.17-1.60)	2.52 (2.19-2.91)
Thyroid disorders (100%)	173	5387	3(2-3)	1.44 (1.23-1.69)	2.68 (2.31-3.11)
Pain (63%), Dyspepsia (37%), and Depression (23%)	155	5184	2 (2-4)	1.43 (1.21-1.69)	2.47 (2.11-2.89)
Asthma (100%), COPD (12%)	161	4742	3 (2-4)	1.66 (1.41-1.95)	2.86 (2.45-3.34)
Pain (100%) and Hypertension (100%)	120	3578	2 (2-3)	1.42 (1.18-1.72)	2.79 (2.33-3.33)
Pain (46%), Osteoporosis (25%), and Dyspepsia (24%)			2 (2-3)	2.00 (1.68-2.37)	3.74 (3.18-4.40)
Men					
No multimorbidity	1408	55 956	1 (0-1)	1 [Reference]	2.12 (2.01-2.23)
Hypertension (100%), Pain (43%), and Dyspepsia (19%)	349	9259	2 (2-3)	1.38 (1.22-1.55)	3.24 (2.92-3.60)
CHD (100%), Hypertension (74%), Stroke (9%)	313	5603	3 (2-4)	1.94 (1.71-2.20)	5.02 (4.49-5.61)
Asthma (100%) COPD (13%) Psoriasis (9%)	173	5051	3 (2-3)	1.32 (1.12-1.54)	2.97 (2.56-3.45)
Pain (54%) Dyspepsia (34%) Prostate disorders (24%)	197	4846	2 (2-3)	1.52 (1.31-1.77)	3.47 (3.02-4.00)
Diabetes (100%), Hypertension (85%) Cancer (100%)	281	4742	3 (2-3)	2.24 (1.97-2.55)	5.28 (4.70-5.94)
	149	4088	2 (2-3)	1.41 (1.19-1.66)	3.35 (2.85-3.93)

Abbreviations: CHD, coronary heart disease; COPD, chronic obstructive pulmonary disease.

^a^ Each cluster was characterized by the 3 health conditions with the highest probabilities greater than 5% of contributing to that cluster, excluding conditions with observed prevalence equal to or less than that of the total population’s expected prevalence. The percentages represent the prevalence of the condition within the cluster, ie, for hypertension, 100% of individuals within the cluster had hypertension.

^b^ All models adjusted for age, ethnicity, education, socioeconomic status and apolipoprotein ε4 status.

^c^ Incidence rate per 1000 person-years.

### Modification by APOE-ε4

**Figure 3.**
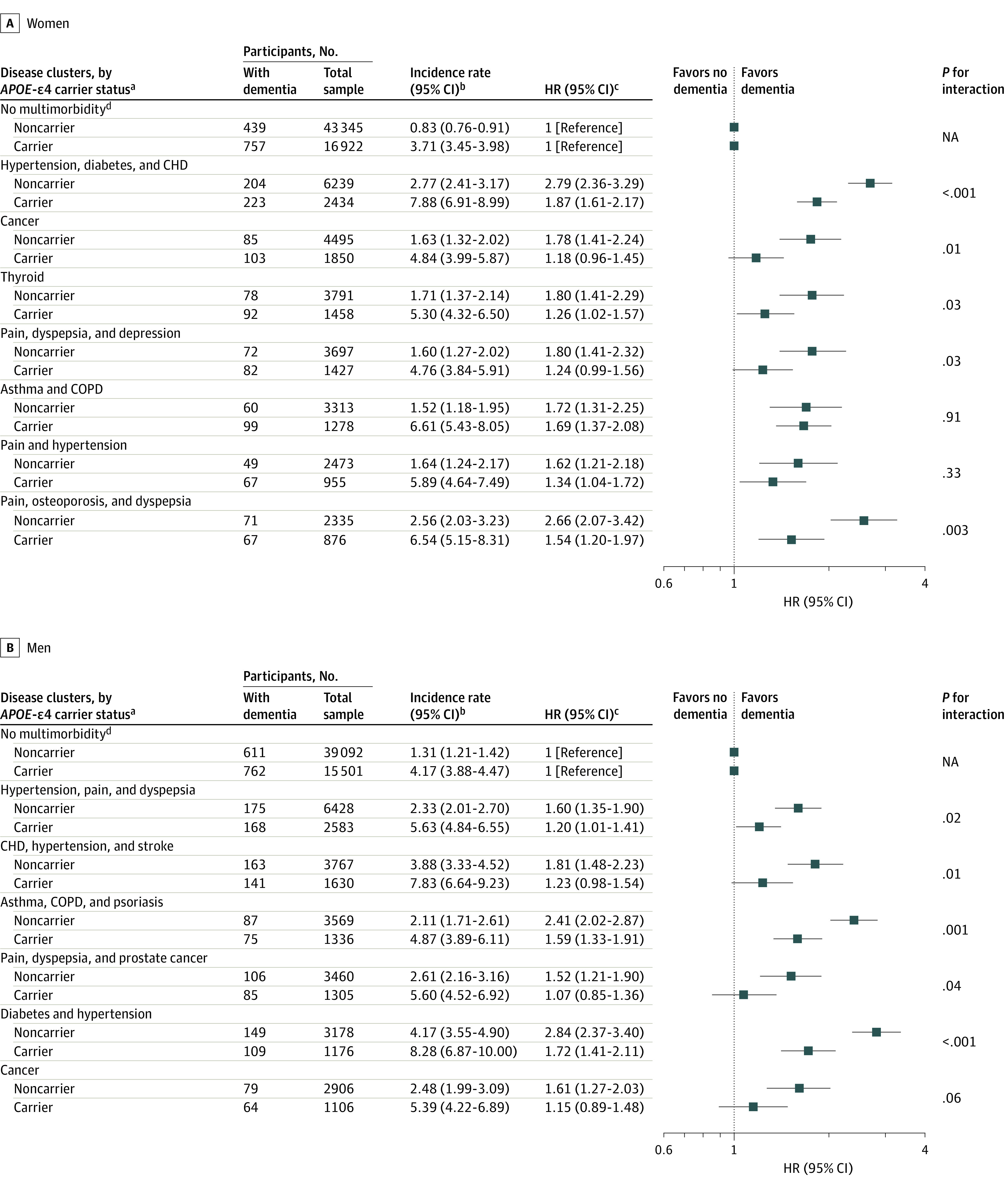
Cox Proportional Hazards Models of the Interaction of Disease Clusters and Apolipoprotein (APOE) ε4 Status With Incident Dementia CHD indicates coronary heart disease; COPD, chronic obstructive pulmonary disease; HR, hazard ratio; NA, not applicable. ^a^Each cluster was characterized by the 3 health conditions with the highest probabilities greater than 5% of contributing to that cluster, excluding conditions for which observed prevalence was equal to or less than that of the total population’s expected prevalence. ^b^Incidence rate per 1000 person-years. ^c^All models adjusted for age, ethnicity, education, socioeconomic status and APOE-ε4. ^d^No multimorbidity non–APOE-ε4 carrier serves as reference group for non–APOE-ε4 clusters and ε4 carrier serves as reference group for ε4 carrier clusters.

Multimorbidity significantly interacted with APOE-ε4 carrier status in association with incident dementia. When stratifying by APOE-ε4 carrier status, the risk of incident dementia associated with multimorbidity was greater in noncarriers (HR, 1.96 [95% CI, 1.81-2.11]) and attenuated in carriers (HR, 1.39 [95% CI, 1.30-1.49]). Similarly, the associations for disease clusters were stronger within noncarriers for both women and men (Figure 3).

However, despite higher relative risks in noncarriers, the absolute risks were higher in APOE-ε4 carriers. For example, among noncarriers, the IR was 2.33 (95% CI, 2.23-2.45) per 1000 person-years for those with multimorbidity, compared with 1.06 (95% CI, 1.00-1.12) per 1000 person-years for those without multimorbidity. In comparison, among APOE-ε4 carriers, the IR was 3.92 (95% CI, 3.73-4.13) per 1000 person-years for those with multimorbidity and 6.12 (95% CI, 5.83-6.41) per 1000 person-years for those without multimorbidity. Therefore, the risk difference, calculated by subtracting the no multimorbidity IRs from the multimorbidity IRs, were higher within APOE-ε4 carriers: 2.20 per 1000 person-years in APOE-ε4 carriers vs 1.27 per 1000 person-years in noncarriers. Similar findings were observed for disease clusters (Figure 3).

## Discussion

In this large UK-based cohort study of more than 200 000 participants aged 60 years or older, multimorbidity was associated with a 63% increased risk of developing dementia over 15 years of follow-up. A dose-response association was observed between number of conditions and dementia risk. Various disease clusters were differentially associated with dementia risk, with hypertension, diabetes, and CHD and pain, osteoporosis, and dyspepsia clusters in women and diabetes and hypertension and CHD, hypertension, and stroke clusters in men associated with double or more the risk of dementia. The associations were consistently stronger among individuals with a lower genetic risk for dementia based on APOE-ε4 carrier status. However, the risk differences between absence and presence of multimorbidity were larger in those with a higher genetic risk for dementia.

Our findings are consistent with a recent study of 10 095 UK-based adults followed over 32 years.^10^ A study by Ben Hassen and colleagues^10^ found that multimorbidity was associated with an increased risk of dementia and that there was a dose-response association between number of conditions and dementia risk. Ben Hassen et al^10^ also observed that the risk of dementia associated with multimorbidity was greater when the onset of multimorbidity was in midlife, with the associations attenuating when multimorbidity occurred at older ages. In this study, we restricted our analyses to participants aged 60 years or older at baseline, since few dementia cases have accrued in participants younger than 60 years. This is likely due to the mean follow-up time of approximately 12 years being insufficiently long for younger participants to develop dementia. In the future, our findings could be extended to investigate whether the associations between particular disease clusters and dementia, as well as the interactions between genetic risk for dementia, vary by age of onset.

To our knowledge, only 1 previous study has explored multimorbidity and different clusters of disease in association with dementia risk. In a study including 2478 adults in Sweden, Grande and colleagues^11^ found that neuropsychiatric, cardiovascular, and sensory impairment or cancer, but not respiratory, metabolic, and musculoskeletal clusters of multimorbidity, were associated with an increased risk of dementia over 12 years follow-up. We identified similar clusters in association with dementia; however, we also found that clusters driven by respiratory and metabolic conditions were associated with dementia risk. Grande and colleagues^11^ included 506 individuals with incident dementia, compared with 6182 individuals with incident dementia included in this study, so they might have lacked the statistical power to detect certain disease clusters in association with dementia.

Grande and colleagues^11^ found no significant interactions for the associations of cardiovascular and neuropsychiatric clusters with dementia risk among APOE-ε4 carriers. In contrast, we found a statistically significant interaction of APOE genotype with multimorbidity and dementia risk, finding stronger associations in noncarriers. The pattern of associations remained similar for disease clusters. Presence of the APOE*-*ε4 allele is responsible for around a quarter of heritability for the most common form of dementia, Alzheimer disease.^24^ APOE*-*ε4 has been reported as associated with an increased risk of various vascular diseases,^25,26^ with inconsistent associations observed for nonvascular diseases, such as depression^27^ and cancer.^28^

Our observation of a higher risk of dementia in noncarriers could be due to the substantially elevated baseline risk of dementia in APOE*-*ε4 carriers, which could attenuate the relative risk of dementia associated with multimorbidity within this group. This is supported by our finding that the absolute risk differences between no multimorbidity and multimorbidity presence and clusters were greater in participants with a higher genetic risk of dementia. Nevertheless, this finding could have important implications for recruitment into prevention trials for Alzheimer disease, which are increasingly incorporating presence of APOE*-*ε4 alleles into the inclusion criteria.^29^ If a trial is targeting disease clusters for dementia risk reduction, then the inclusion of noncarriers might be recommended.

In this study, the strongest associations with dementia for both sexes were identified for clusters largely driven by cardiometabolic and cardiovascular diseases, such as hypertension, diabetes, coronary heart disease, and stroke. This is consistent with the recommendations from expert panels that suggest targeting these conditions for dementia risk reduction.^4,5^ Such recommendations typically focus on these conditions individually, whereas our findings suggest co-occurrence of these conditions might be especially important. Interestingly, despite depression being considered a key risk factor for dementia,^4,30^ we did not identify any clusters that were largely driven by mental health conditions. However, this might be due to the low prevalence of mental health conditions in our sample, with less than 5% of participants having depression or schizophrenia.

We also identified clusters in women and men for less well-established risk factors, such as respiratory diseases and cancer. Meta-analyses have found that reduced pulmonary function and respiratory disease are associated with dementia incidence and death,^31,32^ with reduced oxygen to the brain resulting in neuronal death as a potential causal mechanism.^33^ Conversely, meta-analyses have largely found that cancer is inversely associated with dementia risk.^34,35^ However, previous findings have been based on cancer alone, whereas we found that cancer in the presence of other diseases was associated with an increased risk of dementia. In this study, other clusters grouped diseases from multiple organ systems, such as pain, osteoporosis, and dyspepsia in women and pain, dyspepsia, and prostate disorders in men. These findings can be viewed as hypothesis-generating, with future studies focusing on whether such clusters of disease could provide new insights into the risk of developing dementia. This study has several strengths, including a large sample size, long follow-up period, and the availability of detailed and diverse data, which enabled both a comprehensive definition of multimorbidity and exploration of genetic interactions.

### Limitations

This study has several limitations. The conditions used to define multimorbidity were self-reported. Incident dementia was ascertained using hospital inpatient and death registry records. While studies have found these to be accurate sources of dementia diagnoses, they will underestimate cases captured in other sources, such as primary care or memory clinics.^17,36^ However, misclassification errors or underreporting are likely to have biased the results toward the null. We investigated the associations with Alzheimer disease and vascular dementia; however, the available medical record data have lower accuracy for identifying dementia subtypes.^17^ The future availability of primary care records should address this limitation. Our findings are limited to a UK-based cohort, which also has evidence of a healthy volunteer bias at recruitment.^14^ Therefore, the replicability of our results should be investigated in other populations that have different demographic structures, patterns of disease prevalence, and health care programs. Due to the observational nature of the study, causality cannot be inferred and residual confounding remains.

## Conclusions

This cohort study found that multimorbidity was associated with increased risk of incident dementia. Dementia risk was highest in women with hypertension, diabetes, and CHD or with pain, osteoporosis, and dyspepsia disease clusters and in men with diabetes and hypertension or with CHD, hypertension, and stroke disease clusters. The relative risk was greater in participants with a lower genetic risk of dementia. However, the absolute difference in risk between participants with and without multimorbidity was greater among individuals with a higher genetic risk of dementia. Overall, these findings could improve identification of individuals at high risk of dementia and highlight the necessity of targeting clusters of diseases for dementia prevention rather than individual risk factors.

## Notes

Supplement. **eTable 1.** List of 42 Conditions Used to Define Multimorbidity and Their Prevalence in the Analytic Sample **eTable 2.** *ICD* Codes Used to Ascertain Dementia **eTable 3.** Eight-Class Cluster Solution of Disease Using Latent Class Analysis of Men and Women, Including Sex as a Condition **eTable 4.** Baseline Characteristics of Participants by Incident Dementia **eTable 5.** Cox Proportional Hazards Models for the Association Between Number of Multimorbid Conditions and Incident Dementia **eTable 6.** Cox Proportional Hazards Models of the Interaction of Multimorbidity and Sociodemographic Characteristics With Incident Dementia **eTable 7.** Probabilities and Observed vs Expected Ratios for 41 Conditions Within 7 Clusters in Women **eTable 8.** Probabilities and Observed vs Expected Ratios for 40 Conditions Within 6 Clusters in Men **eTable 9.** Sex-Stratified Cox Proportional Hazards Models for the Association Between Disease Clusters and Incident Dementia in the Test Sample **eFigure.** SABIC Values for Disease Cluster Solutions
