# Supplementary material for: Association of Multimorbidity, Disease Clusters, and Modification by Genetic Factors With Risk of Dementia
Source: JAMA Netw Open. 2022 Sep 20;5(9):e2232124. doi: 10.1001/jamanetworkopen.2022.32124 (PMC9490497; doi:10.1001/jamanetworkopen.2022.32124)
Supplement: Supplement. — eTable 1. List of 42 Conditions Used to Define Multimorbidity and Their Prevalence in the Analytic Sample eTable 2. ICD Codes Used to Ascertain Dementia eTable 3. Eight-Class Cluster Solution of Disease Using Latent Class Analysis of Men and Women, Including Sex as a Condition eTable 4. Baseline Characteristics of Participants by Incident Dementia eTable 5. Cox Proportional Hazards Models for the Association Between Number of Multimorbid Conditions and Incident Dementia eTable 6. Cox Proportional Hazards Models of the Interaction of Multimorbidity and Sociodemographic Characteristics With Incident Dementia eTable 7. Probabilities and Observed vs Expected Ratios for 41 Conditions Within 7 Clusters in Women eTable 8. Probabilities and Observed vs Expected Ratios for 40 Conditions Within 6 Clusters in Men eTable 9. Sex-Stratified Cox Proportional Hazards Models for the Association Between Disease Clusters and Incident Dementia in the Test Sample eFigure. SABIC Values for Disease Cluster Solutions [file jamanetwopen-e2232124-s001.pdf]

Supplemental Online Content

Calvin CM, Conroy MC, Moore SF, Kuźma E, Littlejohns TJ. Association of multimorbidity, disease clusters, and modification by genetic factors with risk of dementia. *JAMA Netw Open.* 2022;5(9):e2232124. doi:10.1001/jamanetworkopen.2022.32124

eTable 1. List of 42 Conditions Used to Define Multimorbidity and Their Prevalence in the Analytic Sample

eTable 2. *ICD* Codes Used to Ascertain Dementia

eTable 3. Eight-Class Cluster Solution of Multimorbidity Using Latent Class Analysis of Men and Women, Including Sex as a Condition

eTable 4. Baseline Characteristics of Participants by Incident Dementia

eTable 5. Cox Proportional Hazards Models for the Association Between Number of Multimorbid Conditions and Incident Dementia

eTable 6. Cox Proportional Hazards Models of the Interaction of Multimorbidity and Sociodemographic Characteristics With Incident Dementia

eTable 7. Probabilities and Observed vs Expected Ratios for 41 Conditions Within 7 Clusters in Women

eTable 8. Probabilities and Observed vs Expected Ratios for 40 Conditions Within 6 Clusters in Men

eTable 9. Sex-Stratified Cox Proportional Hazards Models for the Association Between Disease Clusters and Incident Dementia in the Test Sample

eFigure. SABIC Values for Disease Cluster Solutions

This supplemental material has been provided by the authors to give readers additional information about their work.

eTable 1. List of 42 Conditions Used to Define Multimorbidity and Their Prevalence in the Analytic Sample

**Condition**	**N (%)**	
hypertension	74,932 (36.2)	
painful condition	44,420 (21.5)	
cancer (any)	22,143 (10.7)	
asthma	22,095 (10.7)	
treated dyspepsia	20,932 (10.1)	
coronary heart disease	15,691 (7.6)	
thyroid disorders	14,784 (7.1)	
diabetes	14,382 (7.0)	
depression	9,707 (4.7)	
psoriasis or eczema	6,614 (3.2)	
prostate disorders	6,456 (3.1)	
rheumatoid arthritis, other inflammatory polyarthropathies & systematic connective tissue disorders	5,667 (2.7)	
stroke and TIA	5,573 (2.7)	
osteoporosis	5,502 (2.7)	
chronic obstructive pulmonary disease	5,037 (2.4)	
migraine	4,926 (2.4)	
irritable bowel syndrome	4,576 (2.2)	
glaucoma	3,646 (1.8)	
diverticular disease of intestine	3,512 (1.7)	
anxiety & other neurotic, stress related & somatoform disorders	3,454 (1.7)	
atrial fibrillation	2,591 (1.3)	
inflammatory bowel disease	1,841 (0.9)	
epilepsy	1,524 (0.7)	
chronic sinusitis	1,314 (0.6)	
endometriosis	1,162 (0.6)	
pernicious anaemia	772 (0.4)	
Meniere’s disease	752 (0.4)	
bronchiectasis	745 (0.4)	
chronic fatigue syndrome	706 (0.3)	
peripheral vascular disease	655 (0.3)	
schizophrenia (and related non-organic psychosis) or bipolar disorder	633 (0.3)	
Parkinson's disease	623 (0.3)	
chronic kidney disease	607 (0.3)	
multiple sclerosis	577 (0.3)	
viral hepatitis	485 (0.2)	
chronic liver disease	441 (0.2)	
heart failure	432 (0.2)	
alcohol problems	245 (0.1)	
treated constipation	228 (0.1)	
polycystic ovary	61 (0.03)	
anorexia or bulimia	56 (0.03)	
other psychoactive substance misuse	19 (0.01)	

© 2022 Calvin CM et al. *JAMA Network Open.*

eTable 2. *ICD* Codes Used to Ascertain Dementia

ICD-9	ICD-10
331.0, 290.4, 331.1, 290.2, 290.3, 291.2, 294.1, 331.2, 331.5	F00, F00.0, F00.1, F00.2, F00.9, G30, G30.0, G30.1, G30.8, G30.9, F01, F01.0, F01.1, F01.2, F01.3, F01.8, F01.9, I67.3, F02.0, G31.0, A81.0, F02, F02.1, F02.2, F02.3, F02.4, F02.8, F03, F05.1, F10.6, G31.1, G31.8

Abbreviations: ICD, International Classification of Disease

© 2022 Calvin CM et al. *JAMA Network Open.*

eTable 3. Eight-Class Cluster Solution of Disease Using Latent Class Analysis of Men and Women, Including Sex as a Condition

Cluster[a]	% of training sample (n = 74,874)	Male probability	Lead condition	Subsidiary condition 1	Subsidiary condition 2
1	13.1	0.0	Pain (54.2%)	Dyspepsia (30.0%)	Hypertension (23.6%)
2	15.3	100	Hypertension (87.8%)	Diabetes (37.7%)	CHD (36.1%)
3	8.8	100	Pain (46.1%)	Hypertension (36.7%)	Dyspepsia (30.8%)
4	8.1	7.0	Hypertension (94.2%)	Diabetes (24.6%)	Dyspepsia (20.9%)
5	19.8	40.5	Cancer (100%)	Hypertension (60.8%)	Pain (26.7%)
6	9.3	45.5	Asthma (100%)	Hypertension (49.6%)	Pain (28.4%)
7	18.9	9.5	Thyroid (100%)	Hypertension (49.1%)	Pain (28.1%)
8	6.9	48.8	Hypertension (100%)	Pain (100%)	Dyspepsia (8.2%)

[a] In 5 out of 8 clusters there is a predominant sex, i.e. probability of men contributing to cluster is either <10% or 100%.

© 2022 Calvin CM et al. *JAMA Network Open.*

eTable 4. Baseline Characteristics of Participants by Incident Dementia

Characteristics, N (%)	No incident dementia (N = 200,778)	Incident dementia (N = 6,182)	Total sample (N = 206,960)
Age in years, mean (SD)	64.1 (2.8)	65.7 (2.7)	64.1 (2.8)
Men	94,754 (47.2)	3,224 (52.2)	97,978 (47.3)
Non-white ethnicity	5,583 (2.8)	206 (3.3)	5,789 (2.8)
Education			
College, university or professional qualification	84,784 (42.2)	2,082 (33.7)	86,866 (41.9)
Secondary school or vocational qualification	61,892 (30.8)	1,787 (28.9)	63,679 (30.8)
No qualification	54,102 (27.0)	2,313 (37.4)	56,415 (27.3)
Socioeconomic status in tertiles			
1 (least deprived)	43,681 (21.8)	1,177 (19.0)	44,858 (21.7)
2	123,189 (61.4)	3,587 (58.0)	126,776 (61.3)
3 (most deprived)	33,908 (16.9)	1,418 (22.9)	35,326 (17.0)
APOE-ε4 carrier	54,902 (27.3)	3,345 (54.1)	58,247 (28.1)
Multimorbidity	85,658 (42.7)	3,543 (57.3)	89,201 (43.1)
Follow-up in years, mean (SD)	11.9 (2.1)	9.2 (2.8)	11.8 (2.2)

Abbreviations: APOE, Apolipoprotein E, N, Number of participants, SD, Standard Deviation.

© 2022 Calvin CM et al. *JAMA Network Open.*

eTable 5. Cox Proportional Hazards Models for the Association Between Number of Multimorbid Conditions and Incident Dementia

	**Number of multimorbidities**					
	**0-1**	**2**	**3**	**4**	**5**	**6**
**Dementia cases**	2,639	1,661	982	533	233	134
**Sample**	117,759	48,883	24,644	10,030	3,687	1,957
**Hazard Ratio (95% CI)**	1 (Reference)	1.41 (1.32-1.50)	1.61 (1.49-1.73)	2.19 (1.99-2.40)	2.59 (2.26-2.96)	3.15 (2.65-3.75)
**Incidence Rate (95% CI)**	1.87 (1.80-1.94)	2.88 (2.75-3.03)	3.43 (3.23-3.66)	4.69 (4.30-5.10)	5.65 (4.97-6.42)	6.37 (5.38-7.54)

© 2022 Calvin CM et al. *JAMA Network Open.*

eTable 6. Cox Proportional Hazards Models of the Interaction of Multimorbidity and Sociodemographic Characteristics With Incident Dementia

	No Multimorbidity				Multimorbidity			
	**Dementia cases**	**Sample**	**Hazard Ratio (95% CI)**	**Incidence Rate (95% CI)[b]**	**Dementia cases**	**Sample**	**Hazard Ratio (95% CI)[a]**	**Incidence Rate (95% CI)[b]**
**Age** (*p-value* for interaction = 0.10)								
<65 years	940	70,488	1 (reference)	1.10 (1.03-1.17)	1,073	45,436	1.77 (1.62-1.93)	2.00 (1.88-2.12)
≥65 years	1,699	47,271	1 (reference)	3.03 (2.89-3.18)	2,470	43,765	1.61 (1.52-1.72)	4.93 (4.74-5.13)
**Sex** (*p-value* for interaction = 0.36)								
Men	1,408	55,956	1 (reference)	2.12 (2.01-2.23)	1,816	42,022	1.59 (1.49-1.71)	3.79 (3.62-3.97)
Women	1,231	61,803	1 (reference)	1.64 (1.55-1.74)	1,727	47,179	1.67 (1.55-1.80)	3.09 (2.94-3.24)
**Ethnicity** (*p-value* for interaction = 0.46)								
White	2,569	114,860	1 (reference)	1.86 (1.79-1.94)	3,407	86,311	1.62 (1.54-1.71)	3.39 (3.28-3.51)
Non-white	70	2,899	1 (reference)	2.05 (1.62-2.59)	136	2,890	1.81 (1.36-2.42)	4.13 (3.49-4.89)
**Education** (*p-value* for interaction = 0.94)								
College or higher	977	52,629	1 (reference)	1.54 (1.45-1.64)	1,105	34,237	1.64 (1.51-1.79)	2.75 (2.59-2.91)
Secondary school or vocational	796	36,834	1 (reference)	1.80 (1.68-1.93)	991	26,845	1.61 (1.46-1.77)	3.17 (2.98-3.38)
No qualification	866	28,296	1 (reference)	2.56 (2.40-2.74)	1,447	28,119	1.63 (1.50-1.78)	4.47 (4.25-4.71)
**Socioeconomic status in tertiles** (*p-value* for interaction = 0.24)								
1 (least deprived)	581	27,304	1 (reference)	1.76 (1.62-1.90)	596	17,554	1.49 (1.33-1.67)	2.87 (2.64-3.10)
2	1,554	73,038	1 (reference)	1.77 (1.69-1.86)	2,033	53,738	1.66 (1.55-1.77)	3.24 (3.10-3.39)
3 (most deprived)	504	17,417	1 (reference)	2.44 (2.24-2.67)	914	17,909	1.68 (1.51-1.88)	4.51 (4.22-4.81)

Abbreviations: CI, Confidence Interval.
[a] All models adjusted for age, ethnicity, education, socioeconomic status and APOE-ε4. Specific covariates are dropped from the model where that covariate is the effect modifier of interest.
[b] Incidence rate per 1,000 person-years

© 2022 Calvin CM et al. *JAMA Network Open.*

eTable 7. Probabilities and Observed vs Expected Ratios for 41 Conditions Within 7 Clusters in Women

		Hypertension, diabetes & CHD		Pain, dyspepsia & depression		Cancer		Thyroid disorders		Pain, osteoporosis & dyspepsia		Asthma & COPD		Pain & hypertension	
**Condition**	**Expected**	**P**	**O/E**	**P**	**O/E**	**P**	**O/E**	**P**	**O/E**	**P**	**O/E**	**P**	**O/E**	**P**	**O/E**
hypertension	0.545	1.000	1.83	0.263	0.48	0.434	0.80	0.504	0.93	0.124	0.23	0.313	0.58	1.000	1.83
depression	0.109	0.091	0.83	0.231	2.12	0.071	0.65	0.071	0.65	0.120	1.10	0.088	0.81	0.028	0.25
painful condition	0.408	0.184	0.45	0.625	1.53	0.309	0.76	0.302	0.74	0.455	1.11	0.351	0.86	1.000	2.45
asthma	0.216	0.181	0.84	0.102	0.47	0.094	0.43	0.092	0.42	0.073	0.34	1.000	4.63	0.059	0.27
coronary heart disease	0.087	0.154	1.77	0.043	0.49	0.042	0.48	0.076	0.88	0.138	1.59	0.059	0.68	0.038	0.44
treated dyspepsia	0.203	0.185	0.91	0.374	1.84	0.123	0.60	0.134	0.66	0.241	1.19	0.172	0.85	0.094	0.46
diabetes	0.103	0.230	2.24	0.020	0.19	0.053	0.51	0.088	0.86	0.117	1.14	0.050	0.48	0.054	0.52
thyroid disorders	0.207	0.078	0.38	0.117	0.57	0.119	0.58	1.000	4.83	0.086	0.42	0.135	0.65	0.000	0.00
rheumatoid arthritis	0.067	0.072	1.08	0.035	0.52	0.034	0.51	0.064	0.96	0.188	2.80	0.055	0.82	0.015	0.23
COPD	0.044	0.039	0.89	0.030	0.68	0.021	0.48	0.022	0.49	0.078	1.77	0.121	2.75	0.004	0.10
anxiety	0.039	0.030	0.77	0.107	2.73	0.022	0.57	0.022	0.57	0.033	0.84	0.018	0.47	0.005	0.12
irritable bowel syndrome	0.062	0.037	0.59	0.191	3.08	0.031	0.49	0.025	0.41	0.039	0.62	0.049	0.79	0.012	0.20
alcohol problems	0.001	0.001	0.76	0.004	3.91	0.001	0.72	0.000	0.00	0.002	1.68	0.001	0.90	0.000	0.00
drug misuse	0	0.000	0.00	0.001	0.00	0.000	0.00	0.000	0.00	0.000	0.00	0.000	0.00	0.000	0.00
treated constipation	0.003	0.002	0.58	0.007	2.45	0.001	0.32	0.003	1.11	0.004	1.40	0.002	0.63	0.000	0.00
stroke and TIA	0.042	0.082	1.94	0.016	0.37	0.023	0.55	0.029	0.70	0.074	1.76	0.015	0.36	0.016	0.39
chronic kidney disease	0.005	0.012	2.40	0.003	0.69	0.004	0.71	0.003	0.52	0.004	0.79	0.002	0.45	0.002	0.46
diverticular disease	0.044	0.041	0.93	0.091	2.08	0.023	0.53	0.021	0.47	0.042	0.95	0.040	0.92	0.025	0.56
atrial fibrillation	0.015	0.023	1.51	0.007	0.48	0.009	0.63	0.017	1.14	0.031	2.08	0.009	0.61	0.004	0.29
peripheral vascular disease	0.005	0.007	1.30	0.005	0.97	0.003	0.54	0.002	0.42	0.018	3.57	0.001	0.29	0.001	0.11
heart failure	0.003	0.004	1.21	0.001	0.27	0.002	0.79	0.003	0.87	0.008	2.57	0.003	0.84	0.001	0.46
glaucoma	0.029	0.032	1.11	0.026	0.88	0.024	0.83	0.021	0.74	0.055	1.91	0.023	0.81	0.012	0.42
epilepsy	0.012	0.010	0.84	0.008	0.64	0.010	0.86	0.011	0.92	0.039	3.29	0.009	0.72	0.000	0.03
schizophrenia	0.006	0.005	0.87	0.005	0.86	0.002	0.27	0.010	1.64	0.012	2.00	0.004	0.62	0.000	0.00
psoriasis or eczema	0.056	0.042	0.75	0.089	1.58	0.027	0.47	0.029	0.52	0.092	1.64	0.087	1.55	0.015	0.26

© 2022 Calvin CM et al. *JAMA Network Open.*

inflammatory bowel disease	0.016	0.015	0.96	0.010	0.60	0.012	0.76	0.006	0.39	0.051	3.19	0.014	0.89	0.007	0.42
migraine	0.061	0.036	0.59	0.163	2.66	0.044	0.72	0.031	0.51	0.068	1.11	0.037	0.60	0.012	0.19
chronic sinusitis	0.013	0.007	0.56	0.031	2.35	0.009	0.68	0.006	0.46	0.012	0.94	0.020	1.53	0.002	0.13
anorexia or bulimia	0.001	0.001	0.76	0.001	1.39	0.001	0.81	0.000	0.21	0.003	2.50	0.001	1.40	0.000	0.00
bronchiectasis	0.009	0.008	0.84	0.006	0.67	0.005	0.58	0.004	0.40	0.019	2.12	0.023	2.56	0.000	0.04
Parkinson's disease	0.004	0.003	0.75	0.001	0.28	0.003	0.66	0.001	0.34	0.014	3.57	0.004	0.91	0.000	0.00
multiple sclerosis	0.006	0.006	1.04	0.005	0.79	0.005	0.79	0.003	0.51	0.016	2.70	0.004	0.71	0.002	0.27
viral hepatitis	0.004	0.002	0.54	0.009	2.31	0.003	0.70	0.004	0.94	0.003	0.65	0.002	0.60	0.001	0.20
chronic liver disease	0.005	0.003	0.65	0.006	1.10	0.003	0.67	0.004	0.72	0.012	2.48	0.002	0.35	0.002	0.42
osteoporosis	0.081	0.058	0.71	0.054	0.66	0.080	0.99	0.053	0.65	0.251	3.09	0.075	0.93	0.021	0.26
chronic fatigue syndrome	0.001	0.006	6.09	0.026	26.02	0.005	4.80	0.006	6.36	0.005	5.47	0.010	9.56	0.000	0.41
endometriosis	0.02	0.016	0.82	0.041	2.03	0.013	0.65	0.012	0.58	0.024	1.18	0.014	0.68	0.006	0.32
Meniere’s disease	0.008	0.007	0.88	0.012	1.48	0.005	0.61	0.005	0.61	0.015	1.90	0.003	0.33	0.007	0.84
pernicious anaemia	0.011	0.008	0.69	0.005	0.50	0.002	0.21	0.026	2.34	0.027	2.50	0.005	0.50	0.003	0.30
polycystic ovary	0.001	0.001	0.63	0.002	2.13	0.001	0.95	0.002	1.90	0.001	0.56	0.001	0.78	0.001	1.01
cancer	0.202	0.086	0.42	0.057	0.28	1.000	4.95	0.089	0.44	0.065	0.32	0.121	0.60	0.000	0.00

Abbreviations: O/E, Observed/Expected, P, Probability, CHD, Coronary Heart Disease, COPD, Chronic Obstructive Pulmonary Disease, TIA, Transient Ischemic Attack
Orange cells flag O/E > 1; of these the 3 highest probability conditions > 0.10 are marked as blue.

eTable 8. Probabilities and Observed vs Expected Ratios for 40 Conditions Within 6 Clusters in Men

		Hypertension, pain & dyspepsia		Pain, dyspepsia & prostate disorders		CHD, hypertension & stroke		Asthma, COPD & psoriasis		Diabetes & hypertension		Cancer	
**Condition**	**Expected**	**P**	**O/E**	**P**	**O/E**	**P**	**O/E**	**P**	**O/E**	**P**	**O/E**	**P**	**O/E**
hypertension	0.657	1.000	1.522	0.008	0.012	0.737	1.122	0.511	0.778	0.845	1.286	0.614	0.935
depression	0.07	0.071	1.014	0.142	2.029	0.055	0.786	0.055	0.786	0.033	0.471	0.041	0.586
painful condition	0.351	0.431	1.228	0.542	1.544	0.260	0.741	0.264	0.752	0.215	0.613	0.257	0.732
asthma	0.176	0.015	0.085	0.065	0.369	0.074	0.420	1.000	5.682	0.069	0.392	0.000	0.000
coronary heart disease	0.226	0.000	0.000	0.083	0.367	1.000	4.425	0.115	0.509	0.164	0.726	0.154	0.681
treated dyspepsia	0.185	0.194	1.049	0.336	1.816	0.155	0.838	0.151	0.816	0.082	0.443	0.139	0.751
diabetes	0.197	0.040	0.203	0.051	0.259	0.174	0.883	0.079	0.401	1.000	5.076	0.060	0.305
thyroid disorders	0.047	0.049	1.043	0.075	1.596	0.044	0.936	0.031	0.660	0.043	0.915	0.033	0.702
rheumatoid arthritis	0.033	0.037	1.121	0.055	1.667	0.028	0.848	0.032	0.970	0.014	0.424	0.026	0.788
COPD	0.057	0.040	0.702	0.070	1.228	0.054	0.947	0.129	2.263	0.023	0.404	0.033	0.579
anxiety	0.027	0.029	1.074	0.061	2.259	0.016	0.593	0.017	0.630	0.008	0.296	0.017	0.630
irritable bowel syndrome	0.022	0.024	1.091	0.057	2.591	0.009	0.409	0.017	0.773	0.004	0.182	0.012	0.545
alcohol problems	0.004	0.004	1.000	0.008	2.000	0.002	0.500	0.003	0.750	0.000	0.000	0.003	0.750
drug misuse	0.000	0.000	0.000	0.001	0.000	0.000	0.000	0.000	0.000	0.000	0.000	0.000	0.000
treated constipation	0.001	0.001	1.000	0.002	2.000	0.001	1.000	0.001	1.000	0.001	1.000	0.002	2.000
stroke and TIA	0.07	0.088	1.257	0.072	1.029	0.091	1.300	0.036	0.514	0.056	0.800	0.051	0.729
chronic kidney disease	0.007	0.010	1.429	0.004	0.571	0.010	1.429	0.003	0.429	0.007	1.000	0.008	1.143
diverticular disease	0.023	0.026	1.130	0.042	1.826	0.014	0.609	0.020	0.870	0.013	0.565	0.018	0.783
atrial fibrillation	0.034	0.042	1.235	0.054	1.588	0.028	0.824	0.018	0.529	0.018	0.529	0.028	0.824
peripheral vascular disease	0.007	0.007	1.000	0.010	1.429	0.011	1.571	0.004	0.571	0.007	1.000	0.004	0.571
heart failure	0.006	0.006	1.000	0.007	1.167	0.008	1.333	0.004	0.667	0.004	0.667	0.002	0.333
prostate disorders	0.117	0.135	1.154	0.242	2.068	0.073	0.624	0.081	0.692	0.048	0.410	0.070	0.598
glaucoma	0.035	0.040	1.143	0.056	1.600	0.024	0.686	0.025	0.714	0.025	0.714	0.030	0.857
epilepsy	0.014	0.014	1.000	0.028	2.000	0.011	0.786	0.007	0.500	0.006	0.429	0.012	0.857
schizophrenia	0.006	0.006	1.000	0.017	2.833	0.003	0.500	0.004	0.667	0.004	0.667	0.002	0.333

© 2022 Calvin CM et al. *JAMA Network Open.*

psoriasis or eczema	0.063	0.060	0.952	0.113	1.794	0.040	0.635	0.088	1.397	0.027	0.429	0.035	0.556
inflammatory bowel disease	0.016	0.014	0.875	0.030	1.875	0.011	0.688	0.015	0.938	0.010	0.625	0.013	0.813
migraine	0.022	0.025	1.136	0.058	2.636	0.008	0.364	0.015	0.682	0.002	0.091	0.015	0.682
chronic sinusitis	0.011	0.013	1.182	0.024	2.182	0.003	0.273	0.010	0.909	0.003	0.273	0.004	0.364
anorexia or bulimia	0	0.000	0.000	0.000	0.000	0.000	0.000	0.000	0.000	0.000	0.000	0.000	0.000
bronchiectasis	0.005	0.003	0.600	0.007	1.400	0.002	0.400	0.013	2.600	0.001	0.200	0.004	0.800
Parkinson's disease	0.006	0.006	1.000	0.014	2.333	0.005	0.833	0.003	0.500	0.002	0.333	0.007	1.167
multiple sclerosis	0.003	0.003	1.000	0.006	2.000	0.001	0.333	0.001	0.333	0.002	0.667	0.001	0.333
viral hepatitis	0.004	0.003	0.750	0.011	2.750	0.002	0.500	0.002	0.500	0.001	0.250	0.005	1.250
chronic liver disease	0.004	0.003	0.750	0.007	1.750	0.002	0.500	0.001	0.250	0.004	1.000	0.003	0.750
osteoporosis	0.011	0.008	0.727	0.025	2.273	0.008	0.727	0.013	1.182	0.002	0.182	0.011	1.000
chronic fatigue syndrome	0.003	0.003	1.000	0.009	3.000	0.002	0.667	0.002	0.667	0.002	0.667	0.002	0.667
Meniere’s disease	0.006	0.007	1.167	0.013	2.167	0.002	0.333	0.003	0.500	0.002	0.333	0.005	0.833
pernicious anaemia	0.005	0.004	0.800	0.010	2.000	0.003	0.600	0.003	0.600	0.006	1.200	0.003	0.600
cancer	0.157	0.000	0.000	0.056	0.357	0.000	0.000	0.146	0.930	0.070	0.446	1.000	6.369

Abbreviations: O/E, Observed/Expected, P, Probability, COPD, Chronic Obstructive Pulmonary Disease, TIA, Transient Ischemic Attack
Orange cells flag O/E > 1; of these the 3 highest probability conditions > 0.05 are marked as blue.

© 2022 Calvin CM et al. *JAMA Network Open.*

eTable 9. Sex-Stratified Cox Proportional Hazards Models for the Association Between Disease Clusters and Incident Dementia in the Test Sample

**Disease clusters[a]**	**Dementia cases**	**Sample**	**% Participants in cluster**	**Hazard Ratio (95% CI)[b]**	**Incident Rate (95% CI)[c]**
**Female**					
No multimorbidity	1,231	61,803		1 (reference)	1.64 (1.55-1.74)
Hypertension (100%), pain (50%), dyspepsia (22%)	81	2,156	22.9%	1.56 (1.25-1.96)	3.11 (2.50-3.87)
Cancer (100%)	48	1,599	17.0%	1.42 (1.06-1.90)	2.59 (1.95-3.44)
Asthma (100%), COPD (11%), psoriasis (10%)	49	1,457	15.5%	1.60 (1.20-2.13)	2.80 (2.12-3.70)
Pain (61%), dyspepsia (35%), osteoporosis (16%)	47	1,416	15.0%	1.58 (1.18-2.12)	2.78 (2.09-3.70)
Thyroid disorders (100%)	28	1,062	11.3%	1.16 (0.80-1.69)	2.21 (1.52-3.20)
Hypertension (89%), diabetes (47%), CHD (24%)	53	1,040	11.0%	2.10 (1.59-2.77)	4.41 (3.37-5.78)
Depression (100%), pain (41%), anxiety (14%)	26	704	7.5%	1.92 (1.30-2.83)	3.07 (2.09-4.51)
**Male**					
No multimorbidity	1,408	55,956		1 (reference)	2.12 (2.01-2.23)
Hypertension (100%), CHD (28%), dyspepsia (19%)	96	2,269	26.9%	1.54 (1.26-1.90)	3.69 (3.02-4.51)
Pain (100%), hypertension (69%)	67	2,097	24.9%	1.14 (0.89-1.45)	2.76 (2.18-3.51)
Diabetes (100%), hypertension (84%), CHD (28%)	88	1,401	16.6%	2.28 (1.83-2.83)	5.79 (4.70-7.13)
Asthma (100%), psoriasis (12%), COPD (11%)	45	1,265	15.0%	1.34 (1.00-1.80)	3.07 (2.29-4.11)
Dyspepsia (32%), cancer (26%), CHD (25%)	42	954	11.3%	1.55 (1.14-2.11)	3.92 (2.90- 5.30)
Depression (100%), dyspepsia (21%), anxiety (14%)	16	447	5.3%	1.63 (1.00-2.67)	3.23 (1.98-5.28)

Abbreviations: CI, Confidence Interval.

[a] Each cluster was characterised by the three health conditions with the highest probabilities above 5% of contributing to that cluster, excluding conditions where their observed prevalence was equal to or less than that of the total population of men and women: expected prevalence.

[b] All models adjusted for age, ethnicity, education, socioeconomic status and APOE-ε4

[c] Incidence rate per 1,000 person-years

© 2022 Calvin CM et al. *JAMA Network Open.*

eFigure. SABIC Values for Disease Cluster Solutions

A. Women

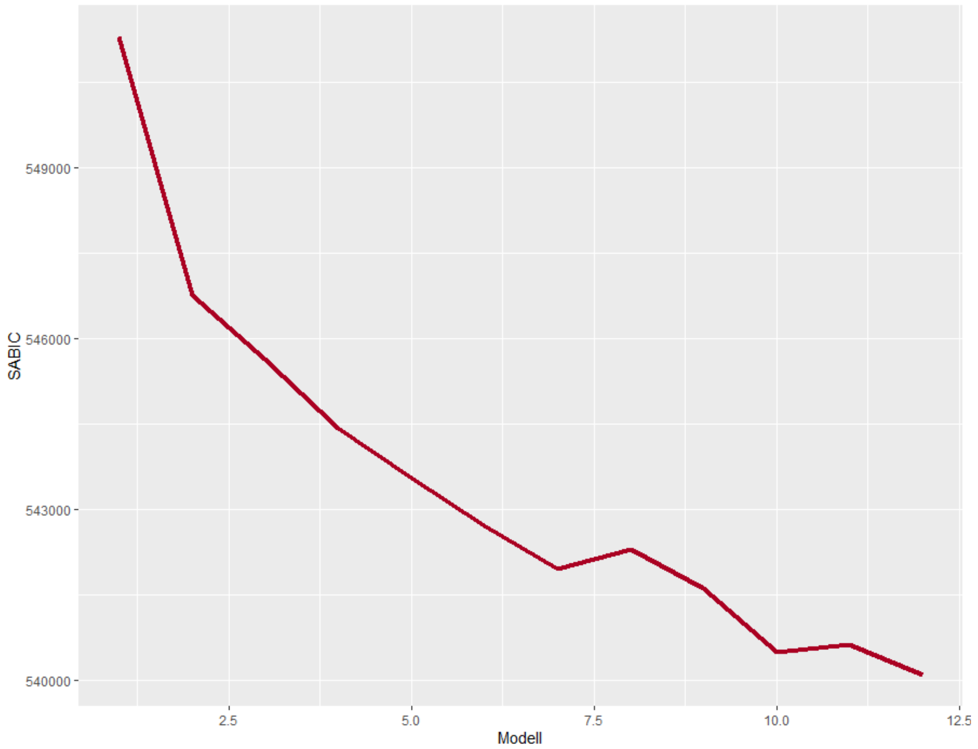

B. Men

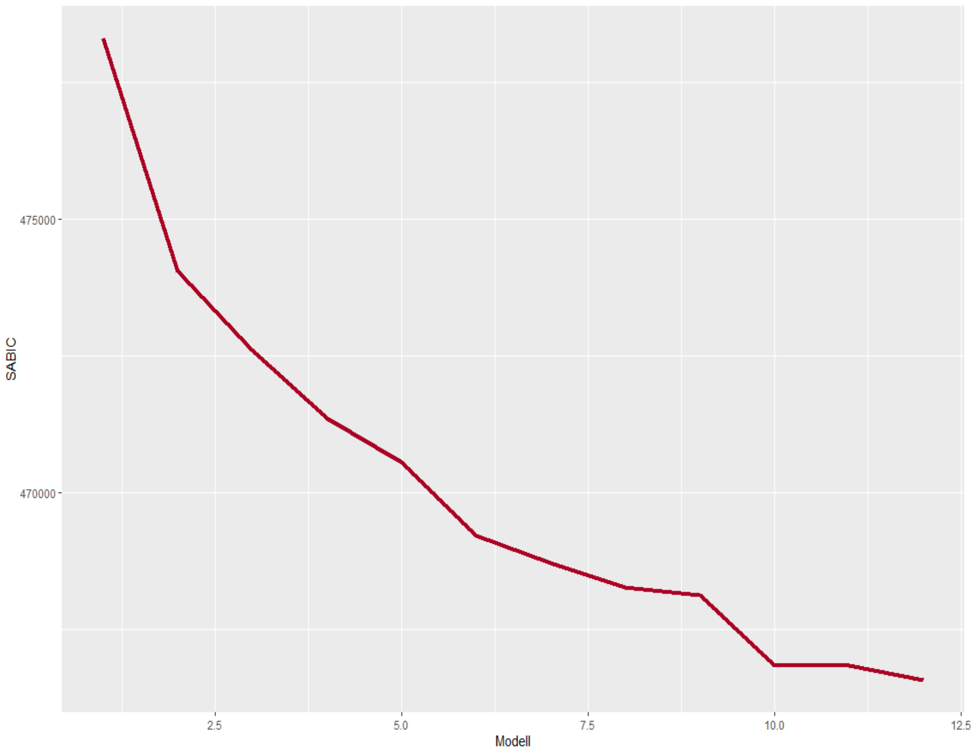

© 2022 Calvin CM et al. *JAMA Network Open.*
